# Search for the role of a Huc-type [NiFe]-hydrogenase of the soil thermophile *Parageobacillus thermoglucosidasius*

**DOI:** 10.3389/fmicb.2026.1754804

**Published:** 2026-02-25

**Authors:** Margarida M. Santana, Jose A. Delgado, Ana Paula Rosa, Cristina Cruz, Juan M. Gonzalez

**Affiliations:** 1 Centre for Ecology, Evolution and Environmental Changes (cE3c) & Global Change and Sustainability Institute (CHANGE), Faculty of Sciences, University of Lisbon, Lisbon, Portugal; 2 Santarém Polytechnic University, School of Agriculture, Quinta do Galinheiro - S. Pedro, Santarém, Portugal; 3 Loyola Biomedical Research Group, Biomedicine and Health Science Department, Faculty of Health Science, Universidad Loyola Andalucía, Dos Hermanas, Spain; 4 Instituto de Recursos Naturales y Agrobiología de Sevilla, Consejo Superior de Investigaciones Científicas, IRNAS-CSIC, Sevilla, Spain

**Keywords:** [NiFe]-hydrogenases, genome analysis, near-zero growth, *Parageobacillus thermoglucosidasius*, persistence in soils, soil thermophilic bacteria

## Abstract

Previously published data showed the ubiquity of thermophilic bacteria in upper soil layers and their potential significant role in biogeochemical cycles. The processes for the maintenance of cell viability by these thermophiles in soils, including cool temperate soils, are largely unknown. We used culturing systems to mimic and analyze usual environmental growth-limiting conditions and near-zero growth rates, namely those imposed by carbon availability, and common in soils. Our goal was to comprehend how a thermophilic bacterium of the Bacillota Phylum, *Parageobacillus thermoglucosidasius* 23.6, persists and maintains its viability in upper soils. Comparative transcriptomic analysis of *P. thermoglucosidasius* 23.6 at optimum growth rate (2.2 h^−1^), slow growth (0.025 h^−1^ and 0.002 h^−1^) and near-zero growth rate (0.0002 h^−1^) revealed the overexpression of [NiFe]-hydrogenase-encoding genes, specifically of those encoding a putative Huc-type high affinity [NiFe]-hydrogenase, under growth limiting conditions. High affinity [NiFe]-hydrogenases were previously shown to be enzymes yielding energy during carbon starvation and to have a major role in the oxidation of tropospheric H_2_ in soil ecosystems; their activity has been proposed as a major sink for global atmospheric H_2_. The presence and expression of these high affinity [NiFe]-hydrogenase-encoding genes are suggested to represent a widespread strategy of terrestrial bacteria, specifically of soil thermophiles, to stay energized among resource variability or limitation, which could be considered a critical mechanism to maintain viability under growth limiting conditions to ensure long-term persistence in soils.

## 1 Introduction

Thermophilic bacteria (including *in sensu stricto* thermophiles, with Tmax > 55 °C and Tmin >30 °C) are common inhabitants of soils (Marchant et al., 2002a; Portillo et al., 2012; Santana and Gonzalez, 2015). Soil thermophilic bacteria (STB) of the Bacillota Phylum (e.g., genera *Geobacillus* and *Parageobacillus*) are ubiquitous in upper soil layers (Marchant et al., 2002a, 2002b) where they remain present as viable bacteria (Marchant et al., 2002a; Portillo et al., 2012). A balance between near-zero growth and negligible death rates has been hypothesized to explain the maintenance of these thermophiles in cool temperate soils (Marchant et al., 2008). At medium and low latitudes, as in the case of the Mediterranean region, where high temperature events on surface soil layers are frequent, with soil temperatures over 40 °C (Portillo et al., 2012), STB have therein temporal opportunities for activity and growth (Portillo et al., 2012; Santana and Gonzalez, 2015). In addition, growth of thermophiles in such non-optimal environments will frequently be carbon-limited, particularly in soils with low content of organic matter and those showing high aridity risk (Gonzalez et al., 2023).

In the Earth’s troposphere, H_2_ is maintained at trace concentrations (0.53 ppmv/0.40 nM) and is rapidly turned over (lifetime ≤ 2.1 y^−1^) (Ehhalt and Rohrer, 2009). Despite the trace concentrations, “tropospheric H_2_ is ubiquitous, unlimited, and energy-rich” (Greening et al., 2014a); the low H_2_ standard redox potential (E°′ = −414 mV) can be employed to reduce all respiratory electron acceptors and its aerobic respiration (H_2_ + ½O_2_ → H_2_O) generates much energy (ΔH° = −276 kJ mol^−1^) (Greening et al., 2022). Thus, H_2_ can constitute a dependable fuel source in soil ecosystems where organic electron donors are often sparse. H_2_ is readily diffusible, so energy-consuming active transport processes are not required. Hence, scavenging atmospheric H_2_ could be a useful strategy for the survival of a range of soil organisms. Indeed, atmospheric H_2_ oxidation by soil ecosystems has been known for over 40 years (Schmidt, 1974; Conrad and Seiler, 1980; Conrad, 1999). Atmospheric H_2_ oxidation is ubiquitous in aerated soils, and it has been observed in a range of soil types and diverse climates (e.g., Smith-Downey et al., 2008). Whole soils take up H_2_ in a biphasic manner, possessing both fast-acting, low-affinity (Km > 800 nM) activities and slow-acting, high-affinity (Km < 70 nM) activities (Häring and Conrad, 1994). [NiFe]-hydrogenases are the enzymes responsible for this uptake, and, in fact, a wide continuum spectrum of affinities for H_2_ is observed in hydrogen-oxidizing organisms (Greening et al., 2014a). Low-affinity [NiFe]-membrane-bound hydrogenases from group 1 [NiFe]-hydrogenases (Vignais and Billoud, 2007), present in H_2_-recycling Proteobacteria, may be responsible for the low-affinity process and used to recycle the relatively high levels of H_2_ produced by biological and geothermal processes (Greening et al., 2015). High affinity hydrogenases (e.g., group 1 h [NiFe]-hydrogenases, Hhy), widespread in soil members of the order Actinomycetales, mediate high-affinity H_2_ oxidation and are major sinks for tropospheric H_2_ in soil ecosystems (Constant et al., 2008; Greening et al., 2014a). This group of hydrogenases is insensitive to oxygen (Schäfer et al., 2013; Greening et al., 2014a), an inactivator of [NiFe] catalytic centers, and thermostable, explaining the observation of atmospheric H_2_ oxidation by soil samples at temperatures ranging from −4 °C to 60 °C (Greening et al., 2015). The fact that high-affinity [NiFe]-hydrogenases can sustain catalytic activity at a wide range of pH values, temperatures, and O_2_ concentrations indicates the robustness and adaptability of these enzymes in the response to environmental shifts. Their activity may thus ensure the energy under deleterious conditions to help bacteria to survive under unstable and/or stressful conditions and contribute to their demonstrated role in long-term viability (Berney and Cook, 2010; Greening et al., 2014b). That might be the case for STB, frequently exposed to stressful environments, in terms of nutrient availability or temperature. Soil thermophilic bacteria might be part of the microbiota oxidizing atmospheric H_2_. Noteworthy, atmospheric H_2_ oxidizers are dominant in a range of cold and hot desert ecosystems worldwide (Greening et al., 2022), the latter are the expected original source of these thermophiles (Marchant et al., 2011).

Considering the gap in knowledge on the maintenance of thermophilic bacteria in soils but the role of high-affinity hydrogenases in cell persistence, our goal was to assess the potential of the thermophilic bacterium *Parageobacillus thermoglucosidasius* 23.6 to scavenge hydrogen, and so to persist in soils, from where it was isolated. We inspected its whole-cell hydrogenase activity, its sequenced genome, the differential expression of genes encoding [NiFe]-hydrogenases, including an identified 2a [NiFe]-hydrogenase (Huc-type hydrogenase), comparing exponential (optimum) growth and growth limiting conditions (slow and near-zero growth rates). To our knowledge, this is the first work where the transcription of bacterial [NiFe]-hydrogenases is followed in a continuum including actual near-zero growth rates that closely mimic the nutrient scarcity at *P. thermoglucosidasius* soil habitat.

## 2 Materials and methods

### 2.1 Genome analysis

*P. thermoglucosidasius* 23.6 genome sequences are available from Genbank in Bioproject PRJNA668107. The complete genome sequence of this strain is available under the accession numbers CP063414–CP063417 and was inspected for the presence and organization of putative [NiFe]-hydrogenases-encoding genes. HydDB, a webtool for the structural and functional classification of hydrogenases (Søndergaard et al., 2016) was used to identify the class of [NiFe]-hydrogenases potentially encoded by *P. thermoglucosidasius* strain 23.6.

### 2.2 Measurement of whole-cell hydrogenase activity

*P. thermoglucosidasius* 23.6 was plated from a glycerol stock on non-selective agar medium (Santana et al., 2020) and grown at 50 °C. A single colony was selected and grown to saturation in liquid medium overnight at 50 °C. Fresh non-selective medium (Santana et al., 2020) was inoculated with 1% (v/v) from the overnight culture in an Erlenmeyer with five times volume air/volume culture. The culture was incubated at 50 °C, 110 rpm and grown until early stationary phase and its cfu mL^−1^ value was then determined following serial dilution and plating. The culture was divided into 2 mL aliquots, which were centrifuged at ambient temperature at 4,000 × g (Spectrafuge 16 M Microcentrifuge; Labnet, Edison, NJ, United States). The pellet of each aliquot was resuspended in 50 μL of Tris 20 mM pH 7.5 after determination of the fresh weight (FW). The cell suspensions were kept at −20 °C and defrost for the hydrogenase activity assay, which consisted of a protocol adapted from Lacasse et al. (2019). Briefly, dilutions of the cell suspensions were performed in 200 μL Tris 20 mM pH7.5 to determine the best cell density for the assay. Triplicates of the dilutions and of negative control (200 μL Tris 20 mM pH7.5) were placed in the wells of a Deltalab 96-well plate (code 900011) and the OD at 630 nm was recorded (MicroPlate reader Synergy HTX, BioTek). The plate was kept briefly in the dark and 20 μL of a freshly prepared developing solution −10 mg/mL benzyl viologen dichloride (Sigma-Aldrich, Massachusetts, United States) and 250 mM sodium formate (Riedel-de Häen) in 20 mM Tris buffer pH 7.5 - were added to each well. The change in absorbance at 630 nm, caused by the reduction of benzyl viologen, was recorded every 42 s for 7 min. Readings continued every 10 min for 1 h. Data were converted to ΔAbs·min^−1^ cfu^−1^and ΔAbs·min^−1^ mg^−1^ (FW). The procedure described was repeated a total of three times using independently grown cell suspensions.

### 2.3 Comparative transcriptomic analysis and growth rate

*Parageobacillus thermoglucosidasius* strain 23.6, isolated from an upper soil sample from Southwestern Andalusia (Coria del Rio, Sevilla, Spain) (Gomez et al., 2020, 2021), was grown in Nutrient Broth (BD Difco, Sparks, MD) at 60 °C at different growth rates. Exponential growth under optimum conditions (2.2 h^−1^) was obtained in a bioreactor and this condition was used as a reference for comparison to slow growing cells. Growth rates down to 0.025 h^−1^ were achieved in a chemostat (Gonzalez and Aranda, 2023) under the same conditions. Lower growth rates and near-zero growth rates were obtained in a retentostat culturing system following previous procedures (Boender et al., 2011; Overkamp et al., 2015; Gonzalez and Aranda, 2023). A retentostat differs from a chemostat in that the retentostat is a closed system for the cells and open to medium (Gonzalez and Aranda, 2023). Cells were filtered back to the vessel by tangential flow filtration (Boender et al., 2011; Overkamp et al., 2015) and, thus, cells were slowly accumulated, forced to share the same nutrient resource, which progressively led to slower growth rates (down to *ca*. 10,000-fold lower rates than optimum growth). These nutrient-limiting conditions mimic the expected limitation of bacterial growth in the natural environment. Three different independent cultures for each growth rate were selected for RNA extraction and sequencing. Cells were collected at optimum rate (18 min doubling time), slow rate (chemostat; 1.1 days doubling time) and very slow and near-zero growth rates (retentostat; 14 d and 141 days doubling times) (Gonzalez and Aranda, 2023). Cells were collected by centrifugation at 4 °C for 5 min, RNA extracted (ZymoBIOMICS RNA extraction kit, ZymoResearch Corp., Irvine, CA). The rRNA was removed from the total RNA and sequenced (RNA-seq) using Illumina HiSeq system (BGI Genomics., Shenzhen, China). RNA-seq based comparative transcriptomic analysis between the gene expression at optimum and decreasing growth rates (down to near-zero growth rates) was carried out using the software suite and the bioinformatic protocol described by Pertea et al. (2016). This procedure follows a statistical test that uses a cumulative upper quartile normalization of FPKM abundance estimates according to Paulson et al. (2013). RNA sequencing data are accessible under DDBJ BioProject PRJDB39277, runs DRR797640-DRR797651.

### 2.4 Statistical analysis

Student’s *t*-test (IBM SPSS Statistics 30.0.0.0) was used to compare significant differences (*p* < 0.05) between hydrogenase activity measurements performed at 25 °C and 50 °C.

## 3 Results

### 3.1 *P. thermoglucosidasius* 23.6 encodes three putative [NiFe]-hydrogenases

The soil Actinomycetota *Mycobacterium smegmatis* has been extensively studied regarding its H_2_-scavenging activity and associated hydrogenases. This bacterium has three hydrogenases that belong to distinct phylogenetic groups, −1 h, 2a, and 3b—which have homologs in other actinomycetes (Greening et al., 2014a). Both 1 h hydrogenase (Hhy), considered a typical high-affinity hydrogenase (Km < 100 nM) and 2a, named Huc, are capable to mediate high-affinity H_2_ oxidation, while Huc is also efficient at a wide range of H_2_ concentrations (Greening et al., 2014a). We have clearly identified in *P. thermoglucosidasius* 23.6 genome sequence the genes encoding a Huc-type enzyme among three putative [NiFe]-hydrogenases-encoding genes; the hydrogenase primary sequences were assigned to hydrogenase groups 1d (Hup-type), 2a (Huc-type) and 4a (Hyf-type).

Although not being classed as H_2_-scavenging, having low affinity for hydrogen (Mohammadi et al., 2017), hydrogenases from group 1d are membrane-bound H_2_-uptake enzymes known to be relatively oxygen-tolerant. Hydrogenases of this group mediate the electron input for respiratory reduction of various electron acceptors such as nitrate, sulfate, and fumarate (Mohr et al., 2018) but are in general associated with aerobic respiration and oxygen-tolerant anaerobic respiration in facultative anaerobes (Greening et al., 2016) and may support autotrophic growth (Mohammadi et al., 2017).

Contrary to the above groups, group 4a comprehends oxygen-sensitive, membrane-bound H_2_ evolving enzymes (Mohr et al., 2018). The group 4 [NiFe]-hydrogenases is traditionally associated with a fermentative role, namely with 4a formate hydrogenlyase, which couples the oxidation of formate to CO_2_ and to fermentative evolution of H_2_ (McDowall et al., 2014).

**Figure 1:**
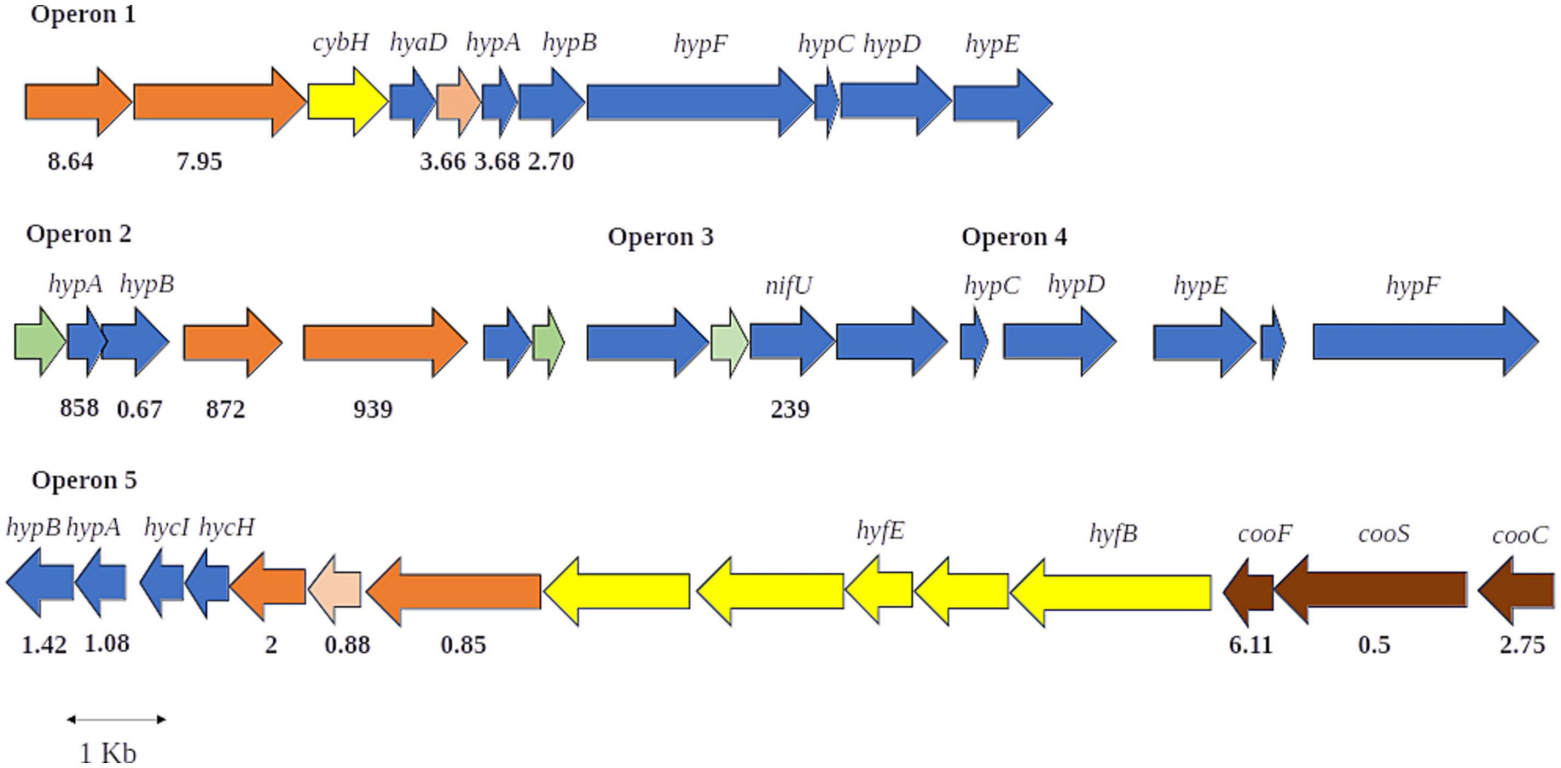
Representation of putative operons 1, 2, and 5 encoding *P. thermoglucodidasius* 23.6 hydrogenases of group 1d (Hup-type), 2a (Huc-type), and 4a (Hyf-type), respectively. Putative operons 3 and 4 with genes encoding maturation factors for synthesis of a functional hydrogenase are also depicted. Genes encoding hydrogenase small and large catalytic subunits are represented by orange arrows, and those annotated as encoding Fe-S binding proteins by pink arrows. The yellow arrows represent genes-encoding *b*-type cytochrome subunit of group 1d hydrogenase and additional subunits of 4a hydrogenase. Genes encoding maturation factors are depicted in blue. CO dehydrogenase genes are shown in brown color. Genes encoding hypothetical proteins are shown in green. The bold numbers under ORFs are the fold change between the gene expression under near-zero growth and optimum growth.

In *Mycobacterium smegmatis*, each of three hydrogenases 1 h (Hhy), 2a (Huc), and 3b, is encoded by a distinct structural operon, and numerous genes encoding maturation factors are clustered with *hyd*1 and *hyd*2 operons encoding, respectively, Huc and Hhy (Greening, 2013). Those maturation factors are required for production of a functional hydrogenase and include, among others, the nickel insertase HypA and the nickel chelator HypB (Greening et al., 2015), required for the nickel insertion into the enzyme (Chan et al., 2012). Like *M. smegmatis*, *P. thermoglucosidasius* 23.6 1d (Hup-type), 2a (Huc-type) and 4a (Hyf-type) hydrogenases are encoded by distinct putative operons (operons 1, 2, and 5; Table 1; Figure 1) and the high similarity of *P. thermoglusosidasius* 23.6 Huc-type hydrogenase to *M. smegmatis* Huc was determined by BlastP analysis of WP_003250668.1 nickel-dependent hydrogenase large subunit (E value = 0). Two additional clusters (operon 3 and 4; Table 1; Figure 1), located in proximity to operon 2, encode maturation proteins, probably used in the assembly of 2a hydrogenase.

**Table 1:** Comparative expression of genes belonging to putative hydrogenase-encoding operons in *Parageobacillus thermoglucosidasius* 23.6 (CP063414.1) over a wide range of growth rates, from optimum growth (maximum rate) down to near-zero growth rate.

		Different growth rates			
Gene ID^a^	Encoded protein	Optimum	Slow growth	Slow growth	Near-zero growth
Doubling time		18 min	1.1 d	14 d	141 d
Growth rate		2.22 h^−1^	0.025 h^−1^	0.002 h^−1^	0.0002 h^−1^
Average FPKM data (SD)^b^					
Operon 1					
IMI45_RS09420	Hydrogenase small subunit	0.89 (0.16)	15.16 (0.27)***	5.89 (0.74)***	7.69 (0.80)***
IMI45_RS09425	Nickel-dependent hydrogenase large subunit	2.06 (0.25)	38.81 (0.22)***	16.51 (10.31)***	16.38 (0.56)***
IMI45_RS09430	Ni/Fe-hydrogenase *b*-type cytochrome subunit	3.93 (0.19)	38.11 (0.31)***	18.11 (1.45)***	14.90 (0.90)***
IMI45_RS09435	HyaD/HybD family hydrogenase maturation endopeptidase	2.29 (0.22)	19.54 (0.75)***	9.48 (1.28)***	7.59 (0.25)***
IMI45_RS09440	Rieske 2Fe-2S domain-containing protein	2.54 (0.06)	24.04 (0.33)***	10.31 (1.89)***	9.30 (0.54)***
IMI45_RS09445	Hydrogenase maturation nickel metallochaperone HypA	2.64 (0.07)	23.38 (0.08)***	11.75 (0.79)***	9.72 (1.50)***
IMI45_RS09450	Hydrogenase nickel incorporation protein HypB	3.95 (0.16)	26.98 (0.50)***	11.40 (0.44)***	10.68 (1.12)***
IMI45_RS09455	Carbamoyltransferase HypF	2.05 (0.07)	13.60 (0.58)***	4.74 (0.13)***	4.99 (0.55)***
IMI45_RS09460	HypC/HybG/HupF family hydrogenase formation chaperone	4.81 (0.13)	23.22 (0.50)***	9.89 (0.71)***	9.93 (0.86)***
IMI45_RS09465	Hydrogenase formation protein HypD	0.11 (0.01)	0.69 (0.12)***	0.89 (0.55)	0.66 (0.22)*
IMI45_RS09470	Hydrogenase expression/formation protein HypE	6.61 (0.24)	26.76 (1.01)***	11.45 (0.93)***	12.03 (1.71)**
Operon 2					
IMI45_RS09585	DUF1641 domain-containing protein	1.91 (0.09)	35.89 (6.56)***	356.37 (17.08)***	713.59 (37.77)***
IMI45_RS09590	Hydrogenase maturation nickel metallochaperone HypA	1.26 (0.05)	35.76 (6.55)***	570.35 (44.41)***	1080.79 (92.03)***
IMI45_RS09595	Hydrogenase nickel incorporation protein HypB	0.03 (0.00)	0.02 (0.00)	0.02 (0.00)	0.02 (0.00)
IMI45_RS09600	Hydrogenase	1.83 (0.05)	49.71 (2.09)***	840.99 (82.30)***	1595.04 (178.84)***
IMI45_RS09605	Nickel-dependent hydrogenase large subunit	1.69 (0.07)	34.95 (1.14)***	762.01 (84.07)***	1586.30 (128.84)***
IMI45_RS09610	Hydrogenase maturation protease	2.21 (0.53)	32.07 (8.82)**	808.12 (80.25)***	1777.36 (170.67)***
IMI45_RS09615	Hypothetical protein	1.26 (0.04)	14.41 (3.56)***	327.98 (40.59)***	660.72 (63.62)***
Operon 3					
IMI45_RS09620	Tetratricopeptide repeat protein	0.03 (0.02)	0.04 (0.00)	0.04 (0.00)	0.04 (0.00)
IMI45_RS09625	Hypothetical protein	2.43 (0.18)	14.68 (0.91)***	293.55 (34.45)***	597.69 (50.27)***
IMI45_RS09630	NifU family protein	1.75 (0.10)	8.85 (2.57)**	196.46 (21.11)***	417.44 (47.83)***
IMI45_RS09635	NHL repeat-containing protein	0.01 (0.00)	0.02 (0.00)	0.02 (0.00)	0.02 (0.00)
Operon 4					
IMI45_RS09640	HypC/HybG/HupF family hydrogenase formation chaperone	0.82 (0.14)	4.74 (1.17)**	120.85 (18.81)***	252.62 (27.40)***
IMI45_RS09645	Hydrogenase formation protein HypD	0.93 (0.11)	4.74 (0.63)***	115.61 (11.13)***	240.43 (17.09)***
IMI45_RS09650	Hydrogenase expression/formation protein HypE	2.84 (0.26)	6.39 (0.22)***	143.50 (16.80)***	292.53 (31.98)***
IMI45_RS09655	Glutathione S-transferase N-terminal domain-containing protein	3.48 (0.13)	6.13 (0.35)***	135.36 (13.02)***	282.53 (23.11)***
IMI45_RS09660	Carbamoyltransferase HypF	5.38 (0.70)	10.00 (0.79)**	48.99 (4.41)***	83.46 (5.30)***
Operon 5					
IMI45_RS10640	Hydrogenase nickel incorporation protein HypB	8.70 (0.71)	14.50 (0.38)***	12.54 (1.69)	12.34 (0.09)***
IMI45_RS10645	Hydrogenase maturation nickel metallochaperone HypA	4.62 (0.90)	6.57 (0.87)	5.05 (0.31)	5.01 (0.06)
IMI45_RS10650	Hydrogenase maturation peptidase HycI	2.70 (0.31)	4.21 (0.26)**	3.34 (0.46)	2.53 (0.37)
IMI45_RS10655	Formate hydrogenlyase maturation HycH family protein	3.31 (0.32)	4.39 (0.20)*	3.54 (0.45)	2.49 (0.39)
IMI45_RS10660	NADH-quinone oxidoreductase subunit B family protein	0.01 (0.00)	0.02 (0.00)	0.02 (0.00)	0.02 (0.00)
IMI45_RS10665	4Fe-4S binding protein	3.44 (0.46)	5.19 (0.68)	4.27 (0.13)	3.01 (0.06)
IMI45_RS10670	Hydrogenase large subunit	2.27 (0.12)	3.52 (0.27)*	2.94 (0.52)	1.94 (0.09)
IMI45_RS10675	Hydrogenase 4 subunit D	1.23 (0.11)	3.02 (0.35)***	2.11 (0.22)**	1.57 (0.23)
IMI45_RS10680	Hydrogenase 4 subunit F	1.29 (0.08)	3.95 (0.55)***	2.33 (0.16)***	2.19 (0.11)
IMI45_RS10685	Hydrogenase 4 membrane subunit	0.99 (0.02)	3.20 (0.41)***	3.04 (0.25)***	2.14 (0.15)***
IMI45_RS10690	Respiratory chain complex I subunit 1 family protein	1.02 (0.22)	3.00 (0.38)***	2.41 (0.11)***	2.30 (0.15)***
IMI45_RS10695	Hydrogenase 4 subunit B	0.96 (0.08)	3.45 (0.66)***	2.57 (0.40)***	2.79 (0.22)***
IMI45_RS10700	4Fe-4S dicluster domain-containing protein	1.40 (0.13)	4.85 (1.50)*	7.99 (0.83)***	8.56 (0.26)***
IMI45_RS10705	CO dehydrogenase, cooS	0.02 (0.00)	0.01 (0.00)	0.01 (0.00)	0.01 (0.00)
IMI45_RS10710	ATPase family protein cooC	3.90 (0.34)	14.28 (0.93)***	9.23 (0.24)***	10.74 (0.50)***

a Genes identified by their order in the genome.

b FPKM, Fragments per kilobase per million mapped reads. Asterisks represent significant difference with respect to optimum growth (*P < 0.05; **P < 0.01; ***P < 0.001).

**Figure 2:**
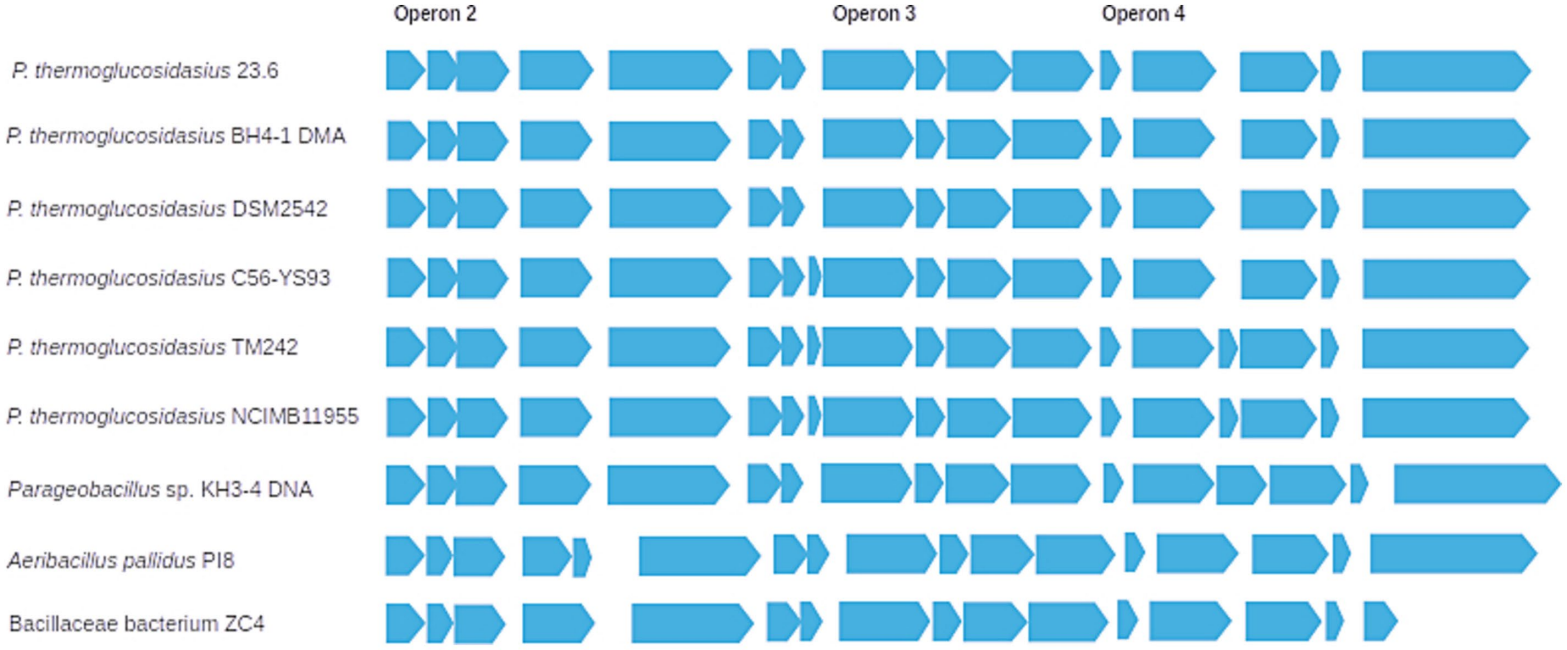
Synteny diagram representing the *loci* associated with the Huc-type [NiFe]-hydrogenase (2a hydrogenase) and the putative operons 3 and 4 encoding maturation proteins in several Bacillaceae members.

The arrangement of the [Ni-Fe] hydrogenase *loci* associated with the 2a hydrogenase in the 23.6 strain (including the putative operons 3 and 4 encoding maturation proteins) is similar to the one already described by Mohr et al. (2018) for *P. thermoglucosidasius* DSM 2542, mostly conserved among several *P. thermoglucosidasius* strains, and roughly retained by other more distant Bacillaceae (Figure 2).

Interestingly, located just upstream of the *P. thermoglucosidasius* DSM 2542 4a hydrogenase *locus*, three putative genes, *cooC*, *cooS*, and *cooF* encoding a CO dehydrogenase maturation factor, a CO dehydrogenase catalytic subunit and a CO dehydrogenase Fe–S protein, respectively, which are involved in the oxidation of CO to CO_2_, were identified leading to the suggestion that 4a hydrogenase may form a complex with a CO dehydrogenase to produce CO_2_ and H_2_. Herein, we also identified putative CO dehydrogenase-encoding genes in the genome of strain 23.6 (see Figure 1; Table 1) indicating the potential of this strain for H_2_ production.

### 3.2 *P. thermoglucosidasius* 23.6 has hydrogenase activity and overexpresses the genes encoding a putative high affinity hydrogenase during limiting growth conditions

**Figure 3:**
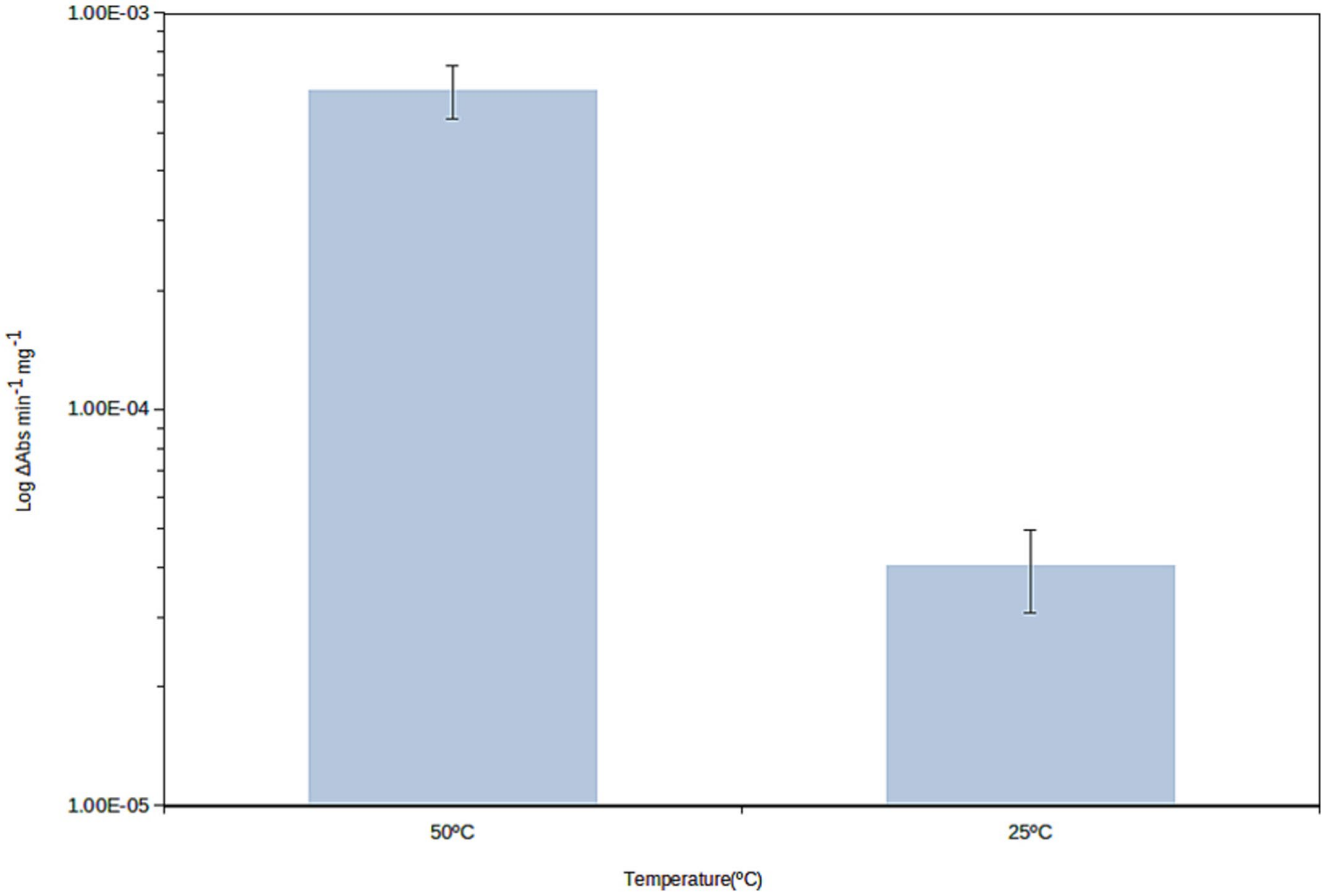
Hydrogenase activity of *Parageobacillus thermoglucosidasius* 23.6 reported to the fresh weight (mg) of cell pellets. Values shown are means of three biological replicates and error bars correspond to standard deviation. Significance was analyzed using a Student’s *t*-test; the difference between groups was significant (*p* < 0.001).

We used benzyl viologen, an established hydrogenase alternative substrate and a dye previously assessed as a colorimetric indicator primarily reporting on [NiFe]-hydrogenase activity (Lacasse et al., 2019). We found hydrogenase activity for cells grown at 50 °C, which was measured at 25 °C and 50 °C. At 25 °C, the activity was (*η* = 3) 4.02 10^−5^ mg^−1^ (FW) (sd = 9.48 10^−6^) and 5.42 10^−11^ cfu^−1^ (sd = 1.53 10^−11^), while at 50 °C the corresponding values were 6.39 10^−4^ mg^−1^ (FW) (sd = 9.86 10^−5^) and 7.49 10^−10^ cfu^−1^ (sd = 1.53 10^−11^). The activity is therefore over 15-fold higher at 50 °C (Figure 3) than at 25 °C although it remains measurable at this low temperature.

Table 1 shows the expression data at different growth rates for the genes within the putative [NiFe]-hydrogenases-encoding operons found in *P. thermoglucosidasius* 23.6. We observed significant overexpression of the genes encoding the subunits of putative [NiFe]-hydrogenases belonging to groups 1d and 2a during limiting growth conditions and located in putative hydrogenase-encoding operons 1 and 2, respectively. 1d (Hup-type) and 2a (Huc-type) nickel-dependent hydrogenase large subunit gene was found to be overexpressed 8-fold and over 900-fold, respectively, at near-zero growth rate compared with optimum growth rate. Decreasing the growth rate to about 89-fold lower than optimum rate indicated important differences, showing overexpression of *hup* and *huc* genes; Huc-type hydrogenase genes were increasingly and drastically overexpressed at decreasing growth rates, reaching maxima at near-zero growth rates, whereas Hup-type enzyme genes were overexpressed at slow growth and near-zero growth, but their expression was highest at a low growth rate (0.025 h^−1^) (Table 1).

Unlike the expression of Hup and Huc [NiFe]-hydrogenase genes, we observed a modest increase in the gene expression of *P. thermoglucosidasius* 23.6 Hyf-type hydrogenase under limiting nutrient conditions versus optimal growth (a 1.6-fold increase of the hydrogenase large subunit gene expression at 0.025 h^−1^).

## 4 Discussion

### 4.1 Predicted sequence-based function of *P. thermoglucosidasius* 23.6 putative [NiFe]-hydrogenases

In most Actinobacteria, enzyme kinetic parameters were determined in whole cells, thus, it is still not clear and not entirely predicted by the primary sequences (Greening et al., 2015), if high-affinity hydrogenases are intrinsically high affinity enzymes or if their affinities are modulated by interactions with the respiratory chain (Greening et al., 2014a). However, recent kinetic analysis of purified *M. smegmatis* Huc enzyme showed that this group 2a enzyme is *per se* capable of oxidizing atmospheric H_2_ with a high affinity (Km = 129 nM) and is highly efficient at low H_2_ concentrations (Grinter et al., 2023). Overall, in addition to the canonical group 1 h [NiFe]-hydrogenase, it has been shown that three other hydrogenase lineages, taxonomically widespread, groups 1f, 1l, and 2a [NiFe]-hydrogenases, can oxidize atmospheric H_2_ (Greening et al., 2014a; Myers and King, 2016; Islam et al., 2020; Ortiz et al., 2020). Since its discovery in Actinomycetota, atmospheric H_2_ oxidation has been shown to be performed by diverse soil organoheterotrophic isolates from phyla Acidobacteriota, Chloroflexota, and Bacteroidota, spanning from thermophiles to psychrophiles (Greening et al., 2022). Globally, bacteria from nine phyla and 17 genera have been experimentally proven to oxidize atmospheric H_2_, and genomic inspections suggest that at least 12 additional phyla contain hydrogenase classes known to intervene in this process (Bay et al., 2021; Ortiz et al., 2020; Greening et al., 2022). One of these phyla is the Bacillota, as *P. thermoglucosidasius* 23.6 genome contains genes putatively encoding a Huc-type enzyme, herein identified through its sequence.

The strain also contains genes encoding putative [NiFe]-hydrogenases of groups 1d (Hup-type) and 4a (Hyf-type). Hup-type hydrogenases, with low H_2_ affinity, are present in a broad range of obligatory aerobic and facultative anaerobic soil-borne, aquatic and host-associated taxa such as *Ralstonia eutropha* and *Escherichia coli* (Alvarez et al., 2010; Greening et al., 2016). In anoxia, 1d O_2_-tolerant hydrogenases may function to keep intracellular O_2_ levels low, thereby protecting O_2_-sensitive enzymes during O_2_ fluctuations (Volbeda et al., 2013). In *P. thermoglucosidasius*, a facultative anaerobe, Hup hydrogenase could have a similar function in anaerobic or microoxic environments. However, it could also be functioning under aerobiosis (see section 4.2.), where it might contribute to hydrogen uptake under elevated H_2_ concentrations, which may be associated with fermentation under moderate carbon availability, favoring mixotrophic growth.

Formate hydrogenlyases, classified as a group 4a [NiFe]-hydrogenases, are widespread in enteric bacteria with a facultatively fermentative lifestyle (Greening et al., 2016). However, group 4 [NiFe]-hydrogenases have been shown to have a respiratory function, as they associate into complexes comprising primary dehydrogenases and terminal hydrogenases to build minimalistic respiratory chains and conserve the energy liberated during electron transfer as a proton or sodium motive force (Buckel and Thauer, 2013). Although minimalistic, these respiratory chains may be of major importance as a primary strategy for energy generation particularly within oligotrophic environments (Greening et al., 2016); these constitute *P. thermoglucosidasius* primary habitat, supporting the relevance of the 4a [NiFe]-hydrogenase in this STB.

Following BlastP analysis to identify [NiFe]-hydrogenases orthologous *loci* in other bacterial taxa, Mohr et al. (2018) referred to the unique combination of [Ni-Fe] group 1- 2a- 4 hydrogenases, which appeared to be restricted to *P. thermoglucosidasius* strains. Orthologous [NiFe] group 2a uptake hydrogenase *loci* were common among the Bacillota, but more restricted to Bacilli and within the family Bacillaceae. As Mohr et al. (2018), we also found orthologous [NiFe] group 2a *loci* among several *P. thermoglucosidasius* strains with similar organization (Figure 2).

The [Ni-Fe] group 4a H_2_-evolving hydrogenase (Hyf-type) *locus* showed the most restricted distribution of the three hydrogenase-encoding *loci* among the Bacillota, with orthologous *loci* only present in *P. thermoglucosidasius* strains and members of the Thermoanaerobacteraceae family (Class Clostridia) (Mohr et al., 2018). In this cases, Hyf-type *loci* organization is similar to that reported for *Caldanaerobacter subterraneus* (Thermoanaerobacteraceae) (Sant’Anna et al., 2015) where 4a H_2_-evolving hydrogenase genes are flanked by three genes, *cooCSF*, coding for a carbon monoxide (CO) dehydrogenase. The *coo* gene cluster was colocalized with the 4a hydrogenase *locus* in nine *P. thermoglucosidasius* genomes inspected by Mohr et al. (2018), but orthologues were not found on the genomes of any other *Parageobacillus* or *Geobacillus* spp. *P. thermoglucosidasius* DSM 2542 was indeed shown to be capable of producing H_2_ when cultured in an initial gas atmosphere consisting of 50% CO and 50% air (Mohr et al., 2018). Herein, we have identified putative CO dehydrogenase-encoding genes in the genome of strain 23.6, adding this strain to the collection of *P. thermoglucosidasius* strains with the potential of CO-dependent H_2_ production.

### 4.2 Predicted physiological role of *P. thermoglucosidasius* 23.6 putative [NiFe]-hydrogenases

In *M. smegmatis*, the expression and activities of 1 h and 2a hydrogenases are significantly increased during carbon limitation; experiments showed that this two H_2_-scavenging enzymes were mostly synthesized and active when the organism growth is severely limited by its preferred organic carbon sources and with highest activity in stationary phase, when organic carbon is scarce (Berney and Cook, 2010; Berney et al., 2014; Greening et al., 2014a; Greening et al., 2022). Genes encoding the structural components of the groups 1 h and 2a were induced 30-fold and 6-fold, respectively, during slow versus fast growth (Berney and Cook, 2010; Berney et al., 2014). Mutant strains lacking these hydrogenases showed a 40% reduction in long-term viability (Berney and Cook, 2010; Greening et al., 2014b). Also, group 1 h hydrogenases show higher activity during energy limitation in other actinomycetes and during sporulation in streptomycetes (Constant et al., 2010; Meredith et al., 2013). In such cases, H_2_ scavenging may function as an electron input to the respiratory chain, H_2_ being the primary electron donor used to maintain redox balance and a membrane potential. Although both Huc and Hhy are oxygen-tolerant, contribute to oxidize H_2_ at sub-atmospheric concentrations, and enhance bacterial survival during carbon limitation, together they enable *M. smegmatis* to oxidize tropospheric H_2_ at significantly faster rates than cultured streptomycetes harboring only group 1 h hydrogenases (Constant et al., 2010; Greening et al., 2014a). Huc and Hhy are indeed differentially expressed, localized, and integrated into the respiratory chain via the menaquinone pool; Hhy is most active during long-term persistence, yielding energy for maintenance processes ensuing carbon exhaustion, whereas Huc is active in late exponential and early stationary phases, “supporting energy conservation during mixotrophic growth and transition into dormancy” (Cordero et al., 2019b). Hence, besides a physiological role in the release of an electron flux to the aerobic respiratory chain that may create sufficient proton-motive force for cells to persist under starvation, hydrogen scavenging is used in *M. smegmatis* for mixotrophic growth with organic carbon sources. The wild-type bacterium appears to be unable to grow chemolithoautotrophically using H_2_ as the sole electron donor (Berney et al., 2014), but the growth rate and yields of deletion strains of group 1 h and 2a [NiFe]-hydrogenases were significantly reduced compared to those of the wild type during growth on organic carbon sources, showing that *M. smegmatis* preferentially grows mixotrophically by co-oxidizing organic electron donors and H_2_ (Berney and Cook, 2010; Berney et al., 2014; Greening et al., 2014b).

More recently, Islam et al. (2020) showed that the group 2a [NiFe]-hydrogenases, moderately to highly abundant in many soils, is largely distributed across several bacterial phyla and can display optimal expression and activity during growth, supporting a mixotrophic growth. In the tested species, belonging to different phylogenetic lineages and ecological niches, the 2a [NiFe]-hydrogenase expression significantly decreased during the transition from growth to persistence, and the enzyme could be used to oxidize H_2_ at sub-atmospheric levels. Hence, such findings demonstrated that “atmospheric H_2_ oxidation is not solely a persistence-linked trait”. Bacteria with 2a [NiFe]-hydrogenases may have selective advantages by co-oxidizing H_2_ with other organic or inorganic energy sources in environments where nutrient availability is very low or variable. In fact, thermodynamic modeling indicates that atmospheric H_2_ used as the sole substrate cannot sustain autotrophic growth, but probably supports mixotrophic growth, especially during growth limitation by organic resources (Conrad, 1999; Bay et al., 2021). In organoheterotrophs, mixotrophic growth may enhance carbon use efficiency by allowing more organic carbon input for anabolism rather than catabolism (Carini, 2021).

Herein, as *P. thermoglucosidasius* 23.6 growth rate decreases down to near-zero growth, cells triggered the increased gene expression of the putative high-affinity type 2a [NiFe]-hydrogenase (Huc-type), which might have a role in a strategy of mixotrophy to compensate for the scarcity of the organic electron donor, hence supporting energy conservation during slow growth. This enzyme also has a potential role in cellular long-term persistence, at near-zero growth of the bacterial population, when it would provide the energy for maintenance processes that follow carbon depletion. Hence, the enzyme could combine the characteristics of *M. smegmatis* Hhy and Huc hydrogenases, confirming the dual role of 2a [NiFe]-hydrogenases indicated by Islam et al. (2020). *P. thermoglucosidasius* 23.6 will gradually increase the expression of the Huc-type enzyme when decreasing cell growth rate, a strategy that would spare cell resources in stressful nutrient-deprived environments and this is certainly related to the extraordinarily adaptability of *P. thermoglucosidasius* to diverse environmental conditions.

In the methanotroph *Methylacidiphilum fumariolicum* SoIV, a hup-type hydrogenase contributed to *M. fumariolicum* autotrophic growth on hydrogen and carbon dioxide, without addition of methane (Mohammadi et al., 2017). In *E. coli*, the 1d hydrogenase shows maximal expression during fermentation when electron acceptors are sparse, but also under several types of stress such as carbon and phosphate starvation, and under stationary phase conditions (Atlung et al., 1997). Likely, the Hup-type hydrogenase of *P. thermoglucosidasius* has a role in energy provision under stress occurring in our experimental case during nutrient-limiting growth (i.e., at slow growth rates), suggesting this enzyme provides the bacterium with an additional mechanism to compete in oligotrophic environments, possibly when local hydrogen levels are high enough due to biological processes that are nevertheless limited by nutrient availability.

In contrast to the expression of Hup and Huc [NiFe]-hydrogenase genes, the increase in *P. thermoglucosidasius* 23.6 Hyf-type hydrogenase gene expression under limiting nutrient conditions is small. *P. thermoglucosidasius* 23.6 could use atmospheric CO as a supplement during carbon starvation, supporting microbial growth and survival, as already demonstrated for *M. smegmatis* (Cordero et al., 2019a) and other Bacillota (Gonzalez and Robb, 2000; Wu et al., 2005; Sant’Anna et al., 2015). Hypothetically, the three *P. thermoglucosidasius* 23.6 [NiFe]-hydrogenases may act cooperatively, with locally evolved H_2_ produced by Hyf being recycled by Hup and Huc-type hydrogenases.

### 4.3 Ecological perspective on atmospheric H_2_ scavenging by *P. thermoglucosidasius*

In soil ecosystems, H_2_ partial pressure (pH_2_) can vary by several orders of magnitude across time and space. For instance, H_2_ partial pressure decreases with soil depth from ambient atmospheric concentrations (530 ppbv) at the surface to threshold levels (<50 ppbv) at about 10 cm depth, the gradient depends on the microbial H_2_ consumption by microbiota (e.g., Smith-Downey et al., 2008). Greening et al. (2015) hypothesized that in zones of high partial pressure of H_2_ (pH_2_), such as those in the vicinity of root nodules due to the H_2_-evolving activity of nitrogenase from N_2_-fixing rhizobacteria, the growth of low-affinity H_2_-oxidizing bacteria (i.e., Alpha-, Beta-, and Gammaproteobacteria harboring group 1 [NiFe]-hydrogenases) is selected (Vignais and Billoud, 2007). In opposite, atmospheric and sub-atmospheric concentrations would support the survival of high-affinity H_2_-oxidizing bacteria, such as the sporulating streptomycetes and persistent mycobacteria. Hence, pH_2_ may be a selective factor for the abundance of copiotrophs versus oligotrophs, since atmospheric H_2_ may confer a dependable lifeline for the latter, and thus influence microbial community structure. As an example, Osborne et al. (2010) demonstrated that exposure of soils to a moderate pH_2_ produced a shift in the soil bacterial community as the relative abundance of ribotypes corresponding to soil actinomycetes *Pseudonocardia*, *Mycobacterium*, and *Streptomyces* species increased. Most importantly, Greening et al. (2015) proposed that atmospheric H_2_ scavenging is important for sustaining the survival of microbes in energy-starved soils by assuring the energy input for basic cell maintenance (i.e., for macromolecular repair, cell wall integrity, membrane potential conservation), environmental sensing and structural changes (e.g., in sporulating cells). Thus, H_2_ scavenging could provide a way to increase cell persistence in the environment under growth limiting conditions, in sum, by generating energy for persisters. In addition, the potential for stress tolerance of high-affinity hydrogenases such as Huc hydrogenase, shown to be thermostable and largely insensitive to inhibition by O_2_ in *M. smegmatis* (Grinter et al., 2023), combined with the ubiquity of atmospheric H_2_ and the abundance of O_2_, would enable scavenging to occur under changing environments and severely growth-limiting conditions, thus contributing to a relative stability of the microbial community structures in soils (Lennon and Jones, 2011) and to the maintenance of the viability of growth-limited microbial species.

Even if STB comprise bacteria of closely related genera (e.g., *Parageobacillus*, *Geobacillus*, *Bacillus*, *Brevibacillus*, *Ureibacillus*), it is compromising to generalize H_2_ scavenging as a strategy to keep cell viability for all, or most STB species. In fact, the above-mentioned studies by Islam et al. (2020) and Mohr et al. (2018) on the distribution of [NiFe]-hydrogenases did not identify the group 2a in the *Geobacillus* genus, although our BlastP search (results not shown) revealed over 90% identity between 2a [NiFe]-hydrogenase of *P. thermoglucosidasius* 23.6 and hydrogenases of other soil thermophilic members (e.g., *Aeribacillus pallidus* previously known as *Geobacillus pallidus*, *Brevibacillus,* and *Bacillus*).

Overall, our work indicates that, besides sporulating streptomycetes and persistent mycobacteria, STB members are part of atmospheric H_2_-scavenging soil microbiota. *P. thermoglucosidasius* populations probably use a putative high-affinity hydrogenase of group 2a in nutrient-limited soils either for slow mixotrophic growth or to persist at near-zero growth. This is in accordance with the source of these thermophiles, transported to cool soils from dust of hot deserts (Marchant et al., 2011). There, they may survive thanks to the activity of high-affinity [NiFe]-hydrogenases for the energy input required for maintenance and to release cytosolic metabolic water, which has been recently theorized to be sufficient to meet hydration needs (Ortiz et al., 2020, 2021). In fact, soil thermophiles such as *P. thermoglucosidasius* 23.6 are some of the best adapted bacteria to thrive under dry conditions (Gonzalez et al., 2023). Some soil thermophiles, including *P. thermoglucosidasius* 23.6, show extracellular enzymes with optimum activity at very dried soil conditions (at water activities below 0.6) (Gomez et al., 2020, 2021). Also, *P. thermoglucosidasius* 23.6 can decompose halogenated pollutants at optimum rates under very dried conditions (water activity around 0.5) in soils (Moxley et al., 2019). It is worth highlighting that STB survive under conditions where the temperature is below their defined temperature growth range (Santana et al., 2020; Milojevic et al., 2022; Gonzalez et al., 2023), namely in the rhizosphere milieu (Rosa et al., 2025), where [NiFe]-hydrogenases could play a fundamental role when these cells drastically reduce their growth rate to near-zero levels. [NiFe]-hydrogenase activity (herein shown to be measurable at 25 °C) and root-exuded metabolites (Rosa et al., 2025) might be sufficient for cell maintenance to assure the persistence of STB in temperate soils.

## 5 Conclusion

*P. thermoglucosidasius* 23.6 encodes three putative [NiFe]-hydrogenases, herein identified through its genome sequence and assigned to hydrogenase groups 1d (Hup-type), 2a (Huc-type) and 4a (Hyf-type). The first two are H_2_-uptake enzymes, while the latter is a H_2_ evolving enzyme. The arrangement of *P. thermoglucosidasius* 23.6 [Ni-Fe] 2a hydrogenase *locus* is similar among several *P. thermoglucosidasius* strains. Whole-cell hydrogenase activity measurements confirmed *P. thermoglucosidasius* hydrogenase activity at 25 °C and 50 °C, being over 15-fold higher at the highest temperature. Transcriptomic analysis showed that Huc-type hydrogenase genes were increasingly and drastically overexpressed at decreasing growth rates, reaching maxima at near-zero growth rates. Previous reports, mentioned above, described 2a [NiFe]-hydrogenases as high-affinity enzymes with a role in atmospheric and sub-atmospheric hydrogen oxidation for energy conservation during mixotrophic growth, conferring selective advantages by co-oxidizing H_2_ with other energy sources in environments where nutrient availability is highly limited. *P. thermoglucosidasius* 23.6 Huc-type enzyme expression strategy would be crucial in stressful nutrient-deprived environments to keep cell viability. Even though our results collectively imply a functional role for a Huc-type hydrogenase in atmospheric hydrogen oxidation and environmental persistence, our evidence is largely transcriptional; a non-specific whole-cell benzyl viologen reduction assay was performed, and further assays are needed to characterize hydrogen uptake and evolution in *P. thermoglucosidasius* and specifically to characterize kinetic parameters of its Huc-type hydrogenase. Also, further genomic and biochemical studies are necessary to assess the ubiquity of high-affinity [NiFe]-hydrogenases among STB. Nevertheless, our work paves the way for exploring the role of [NiFe]-hydrogenases in the persistence and adaptability of STB in a range of environments. This deserves future studies - which will certainly be exciting - especially when assessing adaptability issues to life under drastically extreme conditions of temperature, dryness, and oligotrophy.

## Data Availability

The datasets presented in this study can be found in online repositories. The names of the repository/repositories and accession number(s) can be found in the article/supplementary material.
